# Declining muscle hyperplasia in juvenile trout is associated with a significant impairment of the supportive function of the myogenic progenitor niche

**DOI:** 10.1186/s13395-026-00431-8

**Published:** 2026-06-08

**Authors:** Sabrina Jagot, Nathalie Sabin, Cécile Rallière, Adèle Branthonne, Morgane Chesnais, Cécile Duret, Jérôme Bugeon, Pierre-Yves Rescan, Karl Rouger, Jean-Charles Gabillard

**Affiliations:** 1 INRAE, LPGP, Rennes, 35000 France; 2 Oniris, INRAE, PAnTher, Nantes, 44307 France

**Keywords:** Satellite cells, Muscle-derived cells, Muscle fibers, Pax7, Transplantation, Fish, Hyperplasia, Hypertrophy

## Abstract

**Background:**

Unlike mammals and birds, where new muscle fiber formation (hyperplasia) ceases around birth, large and fast-growing fish such as trout undergo a spectacular post-hatching surge of hyperplasia, followed by a considerably delayed hyperplasia decline. This study investigated the role of muscle stem cells (MuSCs) and their niche in this process by assessing changes in their abundance, myogenic potential and niche functionality.

**Methods:**

Hyperplasia kinetics were investigated by measuring the total number of fibers and their cross-sectional area (CSA) in white muscle across juvenile stages (10 g to 2 kg). Quantification of MuSCs during growth was performed by *pax7 *in situ hybridization. To assess the supportive capacity of the MuSCs niche, muscle-derived cells (MDCs) extracted from the Tg(mlc2:gfp) trout line were transplanted into muscle of wild-type trout at different juvenile stages. Expression of GFP in transplanted muscle was measured as an indicator of myogenic progenitor differentiation.

**Results:**

Histological analysis revealed a significant decrease in hyperplasia and MuSCs density (defined here as *pax7*^+^ cells) between 10 and 500 g trout. Transplantation experiments using MDCs from Tg(mlc2:gfp) trout (10 g donors into 10 g to 2 kg recipients) showed alterations in niche functionality as the trout grew from 10 to 500 g. The transplantation of Tg(mlc2:gfp) MDCs from early to late juvenile donor trout (100 g to 2 kg) into 10 g WT recipients showed a decrease in the GFP signal as the donor weight increased. Detailed analyses of GFP^+^ fibers produced after transplantation showed an enrichment of small-CSA GFP^+^ fibers in 10 g but not 100 g trout recipient muscles, indicating a rapid impairment in niche ability to support hyperplasia. In addition, by comparing trout of the same age but different weights, we demonstrated that weight gain, rather than chronological aging, is a key factor driving this decline.

**Conclusions:**

Overall, these results indicate that the decline in muscle hyperplasia in trout is associated with an early impairment of the MuSC niche, along with a reduced MuSC density. Also, weight gain was found to play a more critical role than aging. These original findings provide new insights into the mechanisms underlying muscle growth as hyperplasia declines in vertebrates.

**Supplementary Information:**

The online version contains supplementary material available at 10.1186/s13395-026-00431-8.

## Background

In amniotes, skeletal muscle is primarily made up of multinucleated muscle fibers, formed mainly during embryonic and fetal development [1]. After birth, the formation of new fibers (hyperplasia) ceases in mouse [2], pig [3], chickens [4], or persists for only a few weeks in rats [5]. In zebrafish, the growth of skeletal muscle during the embryonic and early larval stages is primarily driven by hyperplasia and the hypertrophy of the initial set of fibers [6, 7]. As the larval-to-adult transition begins, the total number of fibers reaches a plateau, and the embryonic fibers are replaced by newly formed fibers in juveniles [8, 9]. In stark contrast, in large and fast-growing fish such as salmonids, a spectacular surge of hyperplasia occurs during juvenile muscle growth, as illustrated by the 200-fold increase in fiber number between hatching and a 4 kg salmon [10]. The hyperplasia decline is also present in these fish but considerably delayed, as the number of muscle fibers is considered to cease when the body length reaches about half its maximum [11]. This highlights a growth pattern in skeletal muscle of fishes that differs markedly from that observed in amniotes. Post-natal muscle hyperplasia requires an active contribution of muscle stem cells (MuSCs) also called satellite cells, which are located between the basal and plasma membranes, defining their anatomical niche [12, 13]. The term of niche refers to the specialized microenvironment that surrounds and regulates the behavior of MuSCs (i.e., quiescence, activation, proliferation, and differentiation). This niche consists of extracellular matrix components, signaling molecules, and interactions with neighboring cells, such as myofibers, fibroblasts, endothelial cells, and immune cells. MuSCs are commonly identified using the well-known Pax7 marker, essential for cell survival, cell fate and self-renewal [14]. Pax7 has also been reported as a MuSC marker in fish, although in zebrafish an additional subtype of myogenic cells lacking Pax7 expression but expressing met (Pax7⁻/met⁺) has been reported [15–18]. Upon activation in the context of growth or injury, these cells generate myogenic progenitors that either fuse to existing muscle fibers to increase their size (hypertrophy) or fuse together to form new muscle fibers (hyperplasia) [19]. With age, exhaustion of MuSCs strongly compromises the muscle regenerative capacity [20, 21]. Similar results have been observed in the context of diseases such as muscular dystrophies [22]. Experimentally, transplantation of MuSCs from young mice produces better muscle regeneration than that with adult MuSCs, reinforcing the hypothesis of a reduction in their intrinsic myogenic potential with aging [23]. Concomitantly, the age of the recipient has been shown to be critical for efficient muscle regeneration, highlighting that the niche, is also essential to promote new fiber formation [24].

Whereas these hallmarks of reduced hyperplasia capacity are evidenced in aged muscle of mammals, the hyperplasia decline in trout is considered to occur during the juvenile stage [25, 26]. The juvenile period is characterized by an early juvenile phase from 5 g to around 500 g (~ 12 month) followed by a late juvenile phase until sexual maturation (1.5—2 kg; 2–3 years). We have previously observed a lower proportion of proliferative MuSCs in 500 g than in 2 g trout and a quiescent state of MDCs in 2 kg trout [27]. Moreover, only a few myofibers were produced after muscle injury in 1.5 kg trout, and muscle regeneration appeared to be impaired, as indicated by the marked accumulation of connective tissue at the injury site [28]. Although these observations were made in a regenerative context, they may indicate a general decrease in myogenic potential during the juvenile growth period.

In the present study, we followed the evolution of hyperplasia during juvenile period of rainbow trout to precisely define its end. The aim of our work was to assess the myogenic progenitor potential and the functionality of their niche in order to define their respective roles in the decline of muscle hyperplasia in trout.

## Methods

### Animals

Immature rainbow trout (*Oncorhynchus mykiss*) were reared in a recirculating rearing system under natural simulated photoperiod and at 12 ± 1 °C. Fish were fed daily ad libitum on a commercial diet. The Tg(mlc2:gfp) trout line was produced to express GFP specifically in the differentiated muscle cells [29].

### Production of 20 g and 1 kg trout of the same age

As a poikilotherm with continuous growth, trout exhibit extraordinary muscle growth plasticity [30]. To obtain 20 g and 1 kg fish at the same age, trout from the same fertilization were fed from the first-feeding stage either 1–2 days/weeks or fed manually ad libitum during 11 months. Three weeks prior to transplantation the restricted trout were fed daily to ensure full growth.

### Muscle-derived cell extraction

For all studies, MDCs were isolated from the dorsal part of the white muscle of Tg(mlc2:gfp) trout as previously described [31]. Briefly, 10 to 80 g of muscle were collected in cold Dulbecco’s modified Eagle’s medium (DMEM; #D7777, Sigma) supplemented with 9 mM NaHCO3, 20 mM HEPES, 15% horse serum, and antibiotics (100 U/ml penicillin, 100 μg/ml streptomycin). Muscle was mechanically dissociated and enzymatically digested by collagenase (#C9891, Sigma) and trypsin (#4799, Sigma) following by successive 100 μm- and 40 μm-filtrations. Cells were resuspended in DMEM containing 10% fetal calf serum (FCS) and 1% antibiotic–antimycotic solution and counted.

### Muscle-derived cell transplantation

Freshly extracted MDCs were maintained on ice until transplantation (< 1 h). To easily locate the transplantation area, 1% of fluorescent red tracer (#F8826, Molecular Probes) was added to the cell suspension. The concentration of the cell suspension was adjusted to 4 × 10^6^ cells/ml to inject 1 × 10^5^ cells to each fish (25 µL). Prior to transplantation, recipient trout were anesthetized with MS-222 at 50 mg/L and placed on a damp cloth. Using a sterile 0.45 mm needle, the cells were transplanted into dorsal white muscle (4 mm depth) behind the dorsal fin and above the lateral line. Three weeks later, fish were euthanized by an overdose of MS-222 (200 mg/L) and muscle samples from transplanted area were taken and split into two parts. Then, both parts were imaged in red (tracer) and green (GFP) fluorescence with a Nikon Multizoom microscope (AZ100). For each image, the area of the red signal above a fixed threshold (i.e., above the background signal) was calculated. Then, surface area of the green signal within the red area was calculated, and the results were normalized by calculating the GFP/Red area ratio. For each fish, control muscle sample from the contralateral part was collected. Finally, muscle samples were fixed for 2 h in 4% paraformaldehyde (PFA) on ice, dehydrated and embedded in low-melting polyester wax (#19,312, Electron microscopy Science).

### Muscle histology

Myotome Sects. (2 mm) were taken from the caudal region (behind the anal fin) of frozen trout weighing between 10 g to 2 kg, using a rotary blade slicer. Images of each section were captured using a camera equipped with a 100 mm lens placed above a light table. Quantification of the white muscle area was performed using Fiji software [32].

Muscle samples were collected in transplanted area from 10 g (11 g; 11 cm; 5 months), 100 g (141 g; 20 cm; 9 months), 500 g (542 g; 31 cm; 17 months), 1 kg (1088 g; 37 cm; 17 months), 1.5 kg (1534 g; 41 cm; 21 months), and 2 kg (2083 g; 44 cm; 21 months) trout and fixed in 4% PFA overnight at 4 °C and embedded in paraffin. Cross-sections were then cut at 7 µm using a microtome (HM355; Microm Microtech, Francheville, France), deparaffinized, rehydrated and stained with Alexa 488-conjugated wheat germ agglutinin (5 µg/mL in PBS; WGA; Molecular Probes # W11261) for 3 h at room temperature, to visualize connective tissue and basal lamina [33]. Samples of transplanted muscle were deparaffinized and rehydrated using serial dilution of ethanol and then permeabilized 3 min in 0.1% Triton X-100/PBS. After three washes, sections were saturated for 1 h with 3% bovine serum albumin, 0.1% Tween 20 in PBS (PBST). Sections were incubated for 3 h with myosin antibody (#MF20, Hybridoma Bank) diluted in blocking buffer. The anti-mouse Alexa 350-conjugated antibody (#A11045, ThermoFisher) was diluted in PBST and applied for 1 h. Sections were mounted with Mowiol containing 4,6-diamidino-2- phenylindole (DAPI; 0.5 μg/mL). All the images were taken with a Nikon digital camera coupled to a Nikon 90i widefield fluorescence microscope.

### In situ hybridization

In situ hybridization was performed on 7 µm-muscle cross-sections using the chromogenic RNAscope® 2.5HD detection reagent RED kit (#322,360; Bio-Techne) according to the manufacturer’s protocol. Briefly, sections were briefly baked at 60 °C for 1 h, deparaffinized and air dried. After 10 min in hydrogen peroxide solution (#322,335, Bio-Techne), sections were treated with 1X Target Retrieval (#322,000; Bio-Techne) for 15 min at 100 °C, followed by 25 min with Protease Plus solution (#322,331, Bio-Techne) at 40 °C. Due to the presence of three pax7 genes in the trout genome [34], a set of probes able (Om-pax7b-cust, # 575,461, Bio-Techne) to recognized *pax7a1*, *pax7a2* and *pax7b* mRNA was previously designed [18]. This set was hybridized for 2 h at 40 °C. All steps at 40 °C were performed in a Bio-Techne HybEZ II hybridization system (#321,720). Nuclei were counterstained with 0.5 µg/mL DAPI and sections were mounted with EcoMount (#EM897L, Biocare medical). In the chromogenic RNAscope assay, the red signal (Fast Red) can be observed with either a white light or fluorescence microscope.

### Automated analysis of images

Fiber size distribution as a function of fish weight was determined on 5 images of the white muscle transversal section for each fish (*n* = 10–12). Cellpose 2 [35] was first used to detect muscle fibers, followed by Fiji [32] to measure the cross-sectional area (CSA) of each fiber.

Quantification of *pax7*^+^ cells was also performed on these images. All DAPI-labelled nuclei were detected with the Fiji stardist plugin [36], then nuclei with an intensity in the red channel greater than 100 were considered *pax7* positive.

The distribution of GFP^+^ and GFP^−^ fibers was obtained using Visilog 6.8 software. Muscle fibers were first detected on the basis of blue channel intensity, then manually corrected in case of erroneous detection. GFP^+^ fiber was determined as a fiber containing more than 50% green pixels after a thresholding above 30 Gy values on the green channel.

The GFP signal on transplanted area was quantified using a macro-command on Fiji software. Basically, the green (GFP) and red (tracer) area above a fixed threshold were quantify and the ratio GFP/Red area was calculated.

### Statistical analyses

Data were analyzed using the non-parametric Kruskal–Wallis rank test followed by the *post-hoc* Dunn test. All analyses were performed with the R statistical package (version 4.3.2).

## Results

### Muscle hyperplasia decreases sharply in early juvenile trout stage

To accurately determine the kinetics of muscle hyperplasia, we calculated the total number of fibers from the early (10 g) to the late (2 kg) juvenile stages. First, we calculated the mean fiber CSA from images of muscle cross-sections labeled with WGA. Next, the myotome area in the caudal part was measured, and the total number of fibers was calculated (Table 1). The mean total fiber number increased 2.3-fold between 10 and 100 g (from 1.0 × 10^5^ to 2.3 × 10^5^ fibers, respectively), corresponding to 4 months of growth. Over the following 8 months, as the weight increased from 100 to 500 g, the total number of fibers increased by about 2.1-fold (5.0 × 10^5^ fibers). Subsequently, the mean total number of fibers stopped increasing and remained around 5.0 × 10^5^ fibers, suggesting that new fiber formation ceased after the weight reached 500 g. Based on these observations, we reasoned that, if the plateau in the total number of fibers resulted from a decline in new fiber formation, then the proportion of small fibers (CSA < 500 µm^2^; diameter < 25 µm) should decrease as the weight of the trout increased. As shown in Supplemental Figures S1A and S1B, the fiber distribution density showed a clear difference in CSA depending on fish weight, with maximum CSA of 7800 µm^2^ (100 µm in diameter) for 10 g trout and 38 000 µm^2^ (220 µm in diameter) for immature 2 kg trout. Consistent with fiber distribution density, the proportion of small fibers decreased from 34 ± 4% to 11 ± 8% in 10 g and 500 g trout, respectively (*p* < 0.01; Supplemental Figure S1C). Thereafter, their proportion, corresponding to less than 6%, remained within the same range among trout weighing 1 kg, 1.5 kg, and 2 kg. To take better account of the huge difference in fiber CSA, the proportion of surface area occupied by the small fibers over the entire area was determined. It represented 4.7%, 0.7% and less than 0.3% in 10 g, 500 g (*p* < 0.001) and larger trout, respectively (Supplemental Figure S1D). Together, the total fiber number and the proportion of small fibers confirmed the sharp decrease in hyperplasia rate in trout during the early juvenile stage (10 g to 500 g).

**Table 1:** Mean total number of white fibers in the myotome reaches a plateau as early as the juvenile stage in trout

Weight class (g)	Mean total number of white fibers (× 10^3^)
10	101.4 ± 20.7^a^
100	235.7 ± 56.2^a^
500	503.3 ± 145.8^b^
1000	440.8 ± 84.0^b^
1500	498.3 ± 77.0^b^
2000	519.8 ± 107.7^b^

Myotome sections were taken from 10 g to 2 kg trout in the caudal region (behind the anal fin), and the white muscle area was calculated. At the same location, cross-sections of the white muscle were stained with Alexa 488-conjugated wheat germ agglutinin (WGA; green) to visualize the extracellular matrix and calculate the mean fiber CSA. For each fish (*n* = 10–12), the total number of white fibers was calculated as follows: fiber number = myotome area/mean fiber area. All statistical significances were calculated using the non-parametric Kruskal–Wallis rank test followed by the post hoc Dunn test. Different letters indicate significant differences between means (means ± SD)

### Density of pax7^+^ cells decreases sharply in muscle at early juvenile trout stage

**Fig. 1:**
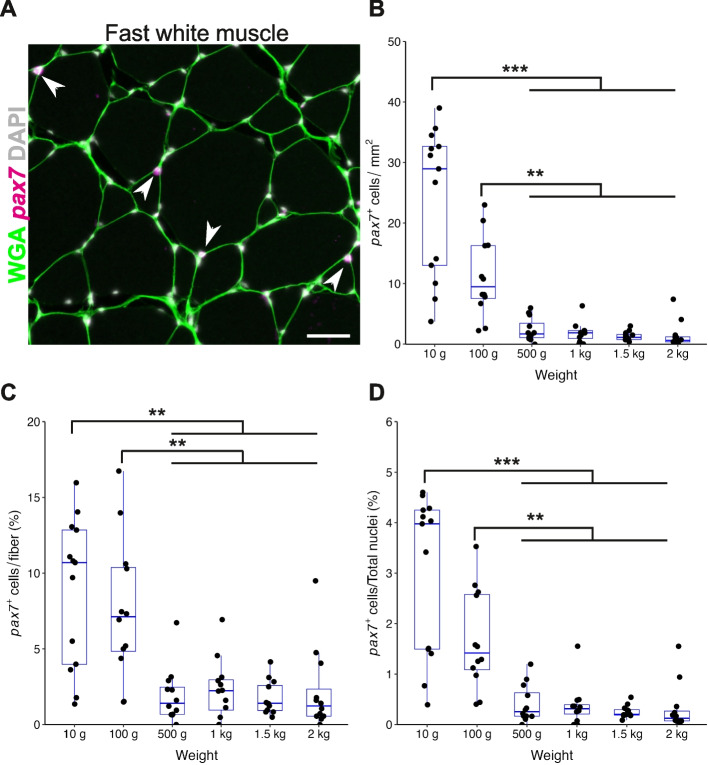
The density of *pax7*^+^ cells in white muscle decreases sharply in early juvenile trout. **A** Cross-sections of trout white muscle were analyzed by in situ hybridization for *pax7* (red) and the extracellular matrix was stained with Alexa 488-conjugated wheat germ agglutinin (WGA; green). Arrowheads indicate *pax7*^+^ cells in the white muscle. The nuclei are counterstained with DAPI and the scale bar corresponds to 100 µm. **B** Quantification of the *pax7*^+^ cells/mm^2^ in white muscle from 10 g to 2 kg trout. **C** Quantification of the percentage of *pax7*^+^ cells/myofiber in white muscle from 10 g to 2 kg trout. **D** Quantification of the percentage of *pax7*.^+^ cells/total nuclei in white muscle from 10 g to 2 kg trout. The data in (**B**), (**C**) and (**D**) are presented as a boxplot (*N* = 8–10). All statistical significances were calculated by the non-parametric Kruskal–Wallis rank test followed by the post-hoc Dunn test. Significance levels: *p < 0.05, **p < 0.01, ***p < 0.001

We recently used a highly sensitive in situ hybridization method to accurately detect the expression of *pax7,* the canonical MuSC marker [18]. Here, this method enabled us to visualize the *pax7*^+^ cells in their typical position under the basal membrane (Fig. 1A) and to count 23.8 ± 3.0 and 2.3 ± 1.5 *pax7*^+^ cells/mm^2^ in muscle section of 10 g and 500 g trout, respectively (Fig. 1B). The density of *pax7*^+^ cells is 10 times higher in the muscle of 10 g trout compared with that of 500 g trout (*p* < 0.001). Thereafter, *pax7*^+^ cells density reached a minimum plateau and remained comparable up to 2 kg trout. As increasing fiber size with fish weight could induce a decrease in *pax7*^+^ cells density, the number of *pax7*^+^ cells/fiber cross-section was then determined (Fig. 1C) showing a decrease as described above. Finally, the density of *pax7*^+^ cells was related to the total number of nuclei counted in a cross-section image of white muscle. Again, the results showed a tenfold reduction (3.0 ± 0.4 *versus* 0.4 ± 0.3; *p* < 0.001) in the proportion of *pax7*^+^ cells between 10 and 500 g juvenile trout (Fig. 1D). Collectively, these data show a change in the density of *pax7*^+^, which falls during the early juvenile stage of trout, leading to low values in trout over 500 g.

### Increasing recipient weight impairs the ability of myogenic progenitors to differentiate

**Fig. 2:**
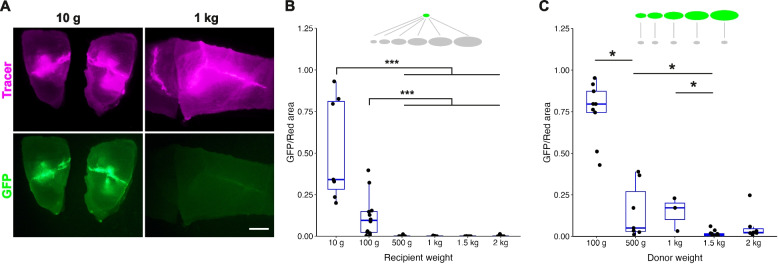
Increasing donor and recipient weight impairs the ability of myogenic progenitors to differentiate. **A** Pictures of muscle samples of 10 g and 1 kg trout, 21 days after transplantation of 1 × 10.^5^ Tg(mlc2:gfp) muscle-derived cells (MDCs) that show the injection site (red tracer) and the endogenous GFP signal (green) resulting from the differentiation of transplanted myogenic progenitors. The scale bar corresponds to 2 mm. **B** Calculation of the ratio between the GFP signal surface and the red surface obtained after transplantation of Tg(mlc2:gfp) MDCs from 10 g trout into muscle of 10 g to 2 kg trout (*N* = 7–12). **C** Calculation of the ratio between the GFP signal surface and the red surface obtained after transplantation of Tg(mlc2:gfp) MDCs from 10 g to 2 kg trout into muscle of 10 g trout (*N* = 7–9, except for 1 kg where only 3 fish could be analyzed). The diagrams in (**B**) and (**C**) represent the transplantation experiment with Tg(mlc2:gfp) donors (green ellipse) into wild-type trout of various sizes (gray ellipse). The data in (**B**) and (**C**) are presented as a boxplot. All statistical significances were calculated by the non-parametric Kruskal–Wallis rank test followed by the post-hoc Dunn test. Significance levels: *p < 0.05, ***p < 0.001

Given the highly dynamic nature of the *pax7*^+^ cell density and the shift from hyperplastic to hypertrophic growth during the juvenile period, we wanted to further explore the impact of trout weight on both the myogenic progenitor potential and the muscle niche functionality. To that end, transplantation assays were performed by using MDCs extracted from Tg(mlc2:gfp) trout line as donor, which allows us to specifically detect differentiated myogenic progenitors thanks to exclusive GFP expression [29]. This was supported by preliminary work on 10 g wild-type (WT) recipients showing that the GFP signal increased up to 29 days post-transplantation and remained stable at least up to 104 days (Supplemental Figure S2). Also, very few CD8^+^ T-cells were identified at the transplantation site in 10 g and 1 kg trout, evoking an absence of a rejection mechanism (Supplemental Figure S3). First, to assess the niche ability to support new fiber formation, Tg(mlc2:gfp) MDCs of donor 10 g trout were transplanted into early to late juvenile recipient trout (10 g to 2 kg). After 21 days, a strong GFP signal throughout the transplantation area was observed in muscle of 10 g trout (Fig. 2A, B), and a clear GFP signal was also observed in the muscle of 100 g trout, but only over a small surface area. Subsequently, a very weak GFP signal was detected in the white muscle of 500 g to 2 kg trout. Additionally, even 60 days after transplantation, no GFP signal was detected in 1 kg wild-type recipients (see Supplemental Figure S2). These data showed that the niche become less and less supportive of myogenic progenitor activity as weight increases, and that beyond 500 g, it is almost no longer functional.

Next, Tg(mlc2:gfp) MDCs from early to late juvenile donor trout (100 g to 2 kg) were transplanted into 10 g WT recipient trout. Twenty-one days later, transplanted MDCs from 100 g donor trout gave a strong GFP signal (Fig. 2C). In comparison, the GFP signal was lower with 500 g donor trout (*p* < 0.05) and almost no longer detectable from 1.5 kg suggesting that the myogenic capacity of MDCs is also linked to weight gain taking into account that we recently reported a 30% decrease of myogenic cells proportion in MDCs between 100 g and 1.5 kg [37]. Taken together, these results suggest that the myogenic capacity of muscle is particularly strong in the early juvenile stage, but this capacity is also rapidly limited by the supportive function of the myogenic progenitor niche. In line with these observations, muscle regeneration capacity assessed by the number of small fibers formed after mechanical injury, was lower in 1 kg trout than in 10 g trout (*p* < 0.001; Supplemental Figure S4).

### The decline in niche functionality occurs prior to the reduction in the myogenic potential of MDCs to form new fibers

**Fig. 3:**
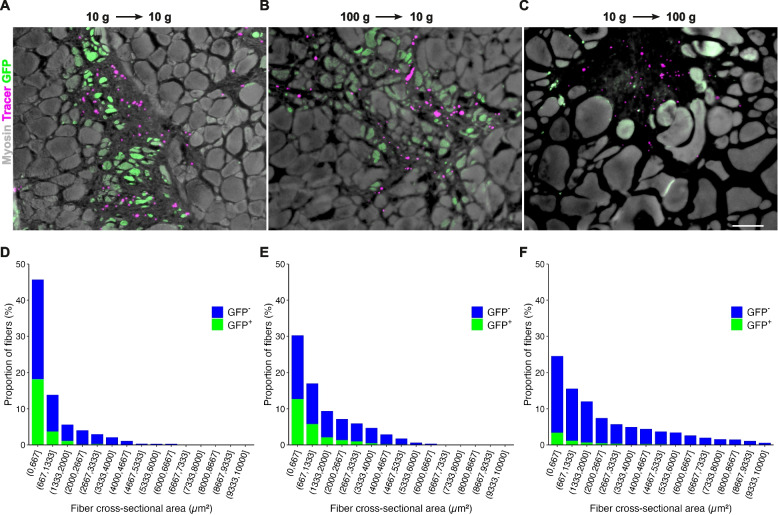
Early decline of muscle hyperplasia potential in trout. Pictures of muscle cross-sections showing immunolabeling for myosin (blue), endogenous GFP (green) and tracer (red) 21 days after transplantation (**A**, **B**, **C**). The scale bar corresponds to 100 µm. Panels A and D correspond to transplantation of 10 g Tg(mlc2:gfp) muscle-derived cells (MDCs) into muscle of 10 g trout. Panels B and E correspond to transplantation of 100 g Tg(mlc2:gfp) MDCs into muscle of 10 g trout. Panels C and F correspond to transplantation of 10 g Tg(mlc2:gfp) MDCs into muscle of 100 g trout. Panels D, E and F correspond to GFP^+^ and GFP^−^ fiber size distribution for CSA up to 10,000 µm.^2^

Knowing that transplanted MDCs contain myogenic progenitors capable of differentiation into recipient muscle after 21 days, we sought to determine in what manner they could participate in hyperplasia or hypertrophy. For that purpose, we determined histologically the size distribution of the GFP^+^ fibers newly formed after 21 days. Muscle cross-sections of 10 g recipient transplanted with Tg(mlc2:gfp) MDCs extracted from 10 and 100 g trout were defined by the presence of numerous GFP^+^ fibers (Fig. 3A, B). An enrichment of GFP^+^ fibers with a CSA < 667 µm^2^ was observed, consistent with the global distribution of the endogenous fiber CSA (Fig. 3D, E). In return, when MDCs from 10 g trout were transplanted into the muscle of 100 g trout, only a few GFP^+^ fibers were detected (Fig. 3C), with also an enrichment of GFP^+^ fibers with a CSA < 667 µm^2^ (Fig. 3F). In our conditions we observed only few GFP^+^ fibers larger than 4000 µm^2^. Due to the autofluorescence observed in trout muscle and the low sensitivity of the GFP signal, we wondered whether the GFP signal would be sufficient to be observed in larger myofibers. Immunostaining on transplanted muscle sections using an antibody directed against GFP, combined with a highly sensitive, background-free DAB revelation method (data not shown) confirmed that larger fibers contain no or few GFP protein. These data suggest that the hyperplastic potential of myogenic progenitors is already present during the earliest stages of growth, and that the muscle niche exerts an inhibitory influence on the expression of this intrinsic capacity from very early on.

### Myogenic potential of MDCs and functionality of progenitor niche decrease with trout weight but not with aging

**Fig. 4:**
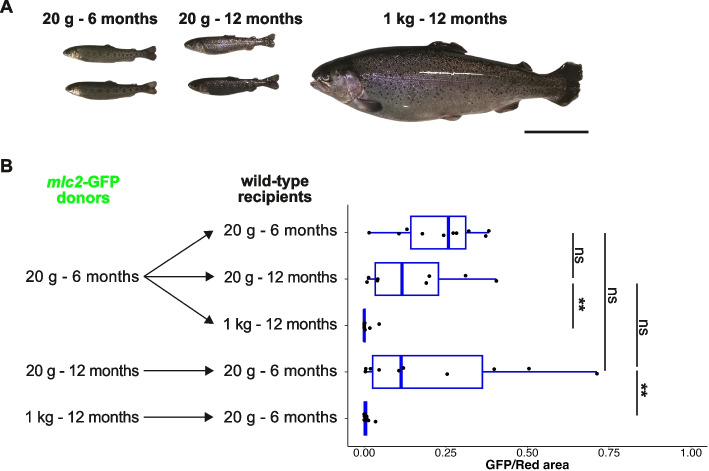
The decline in myogenic capacity of trout muscles is not due to aging but to an increase in weight. **A** Picture of trout for the different combinations of weight/age. The scale bar corresponds to 10 cm. **B** Experimental design of transplantation experiments with different combinations of weight/age trout and calculation of the ratio between the GFP signal surface and the red surface obtained after transplantation of Tg(mlc2:gfp) MDCs into muscle of wild-type trout (*N* = 8–10). All statistical significances were calculated by the non-parametric Kruskal–Wallis rank test followed by the post-hoc Dunn test. Significance levels: **p < 0.01

Considering that trout show exponential growth associated with hyperplastic activity only in the early juvenile stage, we wondered whether increasing weight instead of age might be responsible for the decline in myogenic capacity. To test this hypothesis, we took advantage of the extraordinary plasticity of the trout continuous growth to obtain 20 g and 1 kg fish at the same age (12 months [mo]) by limiting growth through dietary restriction (Fig. 4A). Transplantation of MDCs extracted from 20 g/6 mo trout into 20 g/6 mo and 20 g/12 mo trout resulted in comparable GFP/Red area ratio of 0.23 ± 0.12 and 0.15 ± 0.14, respectively (Fig. 4B). This indicates that the age of the recipient had no major impact on the myogenic capacity of transplanted MDCs. On the other hand, the GFP/Red ratio observed in 1 kg/12 mo trout receiving MDCs from 20 g/6 mo donors was very limited (0.007 ± 0.014), illustrating a significantly lower myogenic potential and pointing to a major effect of the weight status of the recipient on the myogenic progenitor behavior (*p* < 0.01). GFP signals of 0.21 ± 0.24 and 0.007 ± 0.010 were calculated in 20 g/6 mo trout after transplantation of 20 g/12 mo and 1 kg/12 mo trout MDCs, respectively (Fig. 4B). These values show a very reduced myogenic capacity of MDCs from large donors, indicating a negative effect of donor weight gain (*p* < 0.01). Finally, transplantation of MDCs from 20 g/6 mo and 20 g/12 mo trout gave equivalent ratios of 0.23 ± 0.12 and 0.21 ± 0.24 respectively in recipients of 20 g/6 mo, showing no effect of the age of the donor (Fig. 4B). Additionally, the density of fibers (fiber number/mm^2^) and Pax7^+^ cells/mm^2^ are comparable in the 20 g muscle of 6- and 12-month-old trout, but are much higher than in 1 kg trout muscle (see Supplemental Figure S5). Taken together, these data suggest that the myogenic capacity of MDCs is conditioned by the weight of both donor and recipient and not by their age.

## Discussion

The understanding of the muscle hyperplasia process is crucial for deciphering the precise ways in which muscle tissue is formed and to identify the key regulators of MuSCs during myogenesis and muscle repair. These elements are essential with a view to modulating tissue growth and proposing optimized solutions in the context of cell therapies. The availability of a natural biological model such as trout, which exhibits a spectacular post-hatch surge of muscle hyperplasia, offers a unique study context in vertebrates. Here, we sought to define the quantitative and qualitative characteristics of myogenic precursors during juvenile growth, but also to look at the role played by the muscle niche on their potential. The searched aim was to shed light on the reasons for the decline in muscle hyperplasia.

In 1988, a study dedicated to the dynamics of muscle hyperplasia in several fish species concluded that fiber recruitment tends to cease when the body length reaches about half its maximum [11]. However, due to the indeterminate growth of most fish species, this indication cannot be used to accurately estimate the length or weight at which hyperplasia ceases. To determine the kinetics of the hyperplasia, we used two complementary approaches. First, we calculated the total number of fibers by measuring the total muscle area of the caudal section and the mean fiber CSA. Second, we measured the percentage of small fibers (CSA < 500 µm^2^; diameter < 25 µm) as an indicator of new fiber formation [9, 25] throughout the juvenile growth period, although we cannot formally rule out the possibility that these small fibers have not been growing for a long time, or will never grow. Both approaches yielded consistent results, supporting the idea that the hyperplasia rate in white muscle decreases sharply up to 500 g, after which it reaches a minimum plateau. This original result indicates that muscle hyperplasia declines much earlier than previously thought, but remains consistent with earlier work showing that total number of muscle fibers is reached around 1 to 2 kg [26] and that newly formed fibers are still present in white muscle of 1.2 kg trout [25]. Indeed, the fiber size distribution shows the presence of small fibers up to 2 kg, but at very low frequency. The persistence of a small proportion of small fibers, despite no increase in total fiber number in fish over 500 g, may reflect either the replacement of damaged fibers in older animals by newly formed smaller ones, or the presence of fibers that have remained growth-arrested or may never grow. In addition, the negligeable area (< 0.5%) occupied by small fibers in fish weighing over 1 kg, shows that muscle growth is ensured almost exclusively by muscle fiber hypertrophy, with hyperplasia playing a marginal role.

The formation of new muscle fibers after hatching requires the activation and proliferation of MuSCs followed by differentiation and fusion of newly generated myocytes. In this study, Pax7 expression was used to define the MuSC population, while recognizing that this marker does not fully encompass the diversity of myogenic states or their potential transitions. Our thorough quantification of cells expressing the canonical MuSC marker Pax7 revealed a dramatic decrease in their density up to 500 g, followed by a minimal plateau up to 2 kg. This result is in agreement with our previous data showing that proliferative (BrdU^+^) cells in the MuSC niche (beneath basal lamina) were threefold less numerous in white muscle of 500 g than in 2 g trout [27]. Thus, the decrease in MuSC density may result from their lower self-renewal rate. In Zebrafish, density of *pax7*^+^ cells decreases before hatching in white muscle and very rare positive cells are detected on adult [15–17]. In killifish, the number of Pax7^+^ cells per fiber remains relatively stable in adulthood and decreases only in aged fish [38]. In mice, different studies have also reported an age-related decrease in the density of MuSCs [39–42] but differences have recently been observed between muscle types [43]. Indeed, this number decreases with age in locomotor muscles (*Tibialis anterior* and *Extensor digitorum longus*) while it increases in the masseter and remains stable in other muscles. As the trout white muscle is a locomotor muscle, our results corroborate the idea that the MuSC density decline with aging like in equivalent vertebrate muscles.

In trout, our work reveals that the decrease in MuSC density parallels the decline in muscle hyperplasia, since both reach a minimal plateau in trout weighing 500 g, suggesting that the density of MuSCs could partly explain the decline in muscle hyperplasia. Nevertheless, in mouse *Extensor digitorum longus* muscle, MuSC number reaches a minimum plateau 14 days after the cessation of muscle hyperplasia around birth [2], indicating that other parameters are determining as previously suggested [20].

With aging and in the context of muscular dystrophies, it has been shown in mammals that myogenic potential of MuSCs and their niche functionality are impaired [20, 21]. To distinguish the specific role of intrinsic and extrinsic factors of myogenic progenitors in the decline of muscle hyperplasia, transplantation of labeled donor cells into recipient tissue appeared to be the most relevant experimental approach. Our transplantation results indicate that only *Tg(mlc2:gfp)* MDCs from early juvenile trout (< 500 g) were able to differentiate and produce GFP^+^ myofibers, suggesting they preserved their intrinsic myogenic potential. As discussed above, since the density of Pax7^+^ cells decreases with age in trout, one might expect that the lower proportion of GFP^+^ myofibers obtained from MDCs derived from older trout is due to fewer myogenic cells. Because transplanted MDCs are heterogeneous and were not normalized for the number of myogenic progenitors, a decrease in these cells could constitute a confounding factor. For that, our transplantation data do not allow a strict quantification of intrinsic progenitor potential. Nevertheless, we recently performed single cell RNAseq analysis of trout MDCs which revealed only a 30% decrease in the proportion of progenitor cells (MuSCs, myoblasts, and myocytes) among MDCs in fish weighing between 100 g and 1.5 kg [37]. Consequently, the decrease in the proportion of progenitor cells in the transplanted MDCs appears insufficient to fully explain the dramatic 50-fold decline in the myogenic capacity of MDCs as body weight increases. These results are in line with our previous findings indicating that myogenic progenitors exhibit limited in vitro differentiation capacity beyond 1 kg [27], even though they can still produce new muscle fibers at low rate in muscle of 1.5 kg trout [28]. Although we cannot formally rule out the possibility that the observed difference may partly result from a difference in the survival of transplanted cells, we can hypothesize that the decline in myogenic capacity of MDCs results either from a general reduction in the myogenic capacity of progenitors and/or from the loss of a specific myogenic subpopulation required for hyperplasia. Our transplantation results also indicated that MDCs from 10 g *Tg(mlc2:gfp)* trout produced GFP^+^ muscle fibers only in muscle of early juvenile trout recipients, which shows an alteration of the muscle niche functionality as early as late juvenile stage. Moreover, our results highlight that niche alterations that no longer allow the formation of new fibers occur before the decrease in myogenic progenitor potential. These findings are in line with those obtained in rodent, revealing that aged tissue environment impairs myogenic capacities of young MuSCs to form new fibers and to regenerate muscle tissue [24, 44, 45]. In the aged niche of mammals, several authors reported an increase in TGFβ signaling from fibers [44] associated with a decrease in Notch signaling [45] favoring activation and differentiation of MuSCs at the expense of their expansion. In addition, reduction of some component of the extracellular matrix such as fibronectin acts through p38αβ MAPK pathway to make MuSCs non-responsive to FGF2 signaling [46]. It would be interesting in future studies to decipher these pathways in indeterminate growth models such as trout in order to understand whether the mechanisms involved could be comparable.

Overall, this study highlights a significant limitation in the functionality of the myogenic progenitor niche. However, the concurrent decline in the intrinsic capacity of these cells has yet to be definitively confirmed. This is consistent with the low muscle regeneration capacity of 1-kg trout compared to 10-g trout. According to these results, we have already observed few formation of fibers during muscle regeneration of 1.5 kg trout in addition to a strong development of the connective tissue at the injury site [28], a typical landmark of aged muscle [47]. In contrast to trout, adult zebrafish, which exhibit determinate growth, maintain good muscle regeneration capacity without significant connective tissue development [16]. In line with these results, MDCs transplanted in adult zebrafish are still able to produced news fibers evoking that the niche has preserved its functionality [48]. In fish with indeterminate growth, such as sea bream (*Sparus aurata*), almost complete regeneration of muscle has been reported without development of connective tissue [49–51]. However, the fish used in this study were at early juvenile stage (15–25 g) comparable to that of 10 g trout. These results suggest that fish with determinate growth retain a good capacity for muscle regeneration in adulthood, whereas those with indeterminate growth, such as trout or sea bream, see their muscle regeneration capacity declines after the hyperplastic growth period.

Of note, if the decline in myogenic capacity of MuSCs and niche functionality are hallmarks of aging in mammals, in return the juvenile trout of 500 g are young (1 year) and far from sexual maturation (2 years, 2–3 kg). Importantly, the comparable myogenic potential of MDCs observed in 12-month and 6-month-old trout of 20 g demonstrated that this decline is not due to aging but rather to increased weight. In other words, we could hypothesize that the period of exponential muscle growth up to 500 g leads to a significant impairment of the functionality of the myogenic progenitor niche and, potentially, of the intrinsic potential of these progenitors, alongside a dramatic decline in the number of MuSCs. During this period, the high rate of both muscle hyperplasia and hypertrophy requires extensive MuSC contribution, which may exhaust the pool of MuSC and abolishes their self-renewal capacity. These results are reminiscent of those observed in the case of myopathies, where the chronic degeneration processes lead to the MuSC depletion [22]. In contrast, it is interesting to note that fish with determinate growth such as zebrafish exhibit little muscle hyperplasia after hatching, thus preserving the MuSC pool and possibly explaining the efficient muscle regeneration observed in adult. In line with these observations, it has been shown that the number of MDCs extracted from adult fish is inversely related to the muscle mass, with trout having the lowest quantity [52]. For example, *Danio dangila*, a specie closely related to zebrafish (*Danio rerio*) but with indeterminate growth, has 6 times fewer MDCs than zebrafish.

## Conclusions

Our study shows that the decline of muscle hyperplasia in juvenile trout is associated with an early impairment of the niche supportive function. These features, present in some myopathies and muscle aging, may also partly explain the arrest of hyperplasia around birth in mammals and birds. This work underscores the relevance of trout, whose evolution of muscle hyperplasia is highly dynamic, as a model for studying muscle biology and highlights the importance of niche environment in the formation of new muscle fibers in adulthood.

## Supplementary Information

Supplementary Material 1. Supplemental Figure S1: Proportion of small fibers in white muscle decreases sharply in juvenile trout. (A) Cross-sections of white muscle from 10 g to 2 kg trout were stained with Alexa 488-conjugated wheat germ agglutinin (WGA; green) to visualize the extracellular matrix. The nuclei are counterstained with DAPI and the scale bar corresponds to 100 µm. (B) Density function of CSA (µm^2^) in white muscle from 10 g to 2 kg trout. The density values were calculated by normalizing the data so that the sum of the area under the curve equals 1. (C) Percentage of myofibers with a CSA of less than 500 µm^2^. (D) Percentage of the surface occupied by the fibers of less than 500 µm^2^. The data in (C) and (D) are presented as a boxplot (N = 8-10). All statistical significances were calculated by the non-parametric Kruskal–Wallis rank test followed by the post-hoc Dunn test. Significance levels: *p<0.05, **p<0.01, ***p<0.001.

Supplementary Material 2. Supplemental Figure S2: Tg(mlc2:gfp) muscle-derived cells transplanted into wild-type trout were able to differentiate. (A) Tg(mlc2:gfp) muscle-derived cells (MDCs) from 10 g trout were transplanted into muscle of 10 g and 1 kg wild-type trout. For each time point, muscle samples from the transplantation site were collected and pictures were taken to measure red tracer and GFP signal surface using a Nikon multizoom macroscope. Data present the ratio between the GFP and the red surface. (N = 6-10). (B) Data presented as a boxplot showing the GFP/Red Surface ratio obtained after transplanting Tg(mlc2:gfp) MDCs into muscle of 10 g wild-type trout. All statistical significances were calculated by the non-parametric Kruskal–Wallis rank test followed by the post-hoc Dunn test. Bars represent the standard error and the letters indicate the significant differences between means within the same treatment

Supplementary Material 3. Supplemental Figure S3: Transplantation of muscle-derived cells did not induce an immune rejection reaction at the injection site. Cross-sections of trout white muscle were performed 21 days after transplantation and analyzed by *in situ* hybridization for *cd8* (red, marker of T-cells; probe set: Om-Cd8a-C1, # 1199941-C1, Bio-Techne) and the extracellular matrix was stained with Alexa 488-conjugated wheat germ agglutinin (WGA; green). Muscle cross-sections for ISH analysis were first deparaffinized using a serial bath of xylene, then ethanol and finally PBS. Since the red beads are made of polystyrene, they dissolved in the xylene bath and became invisible. No signal was observed with the negative probe against a bacterial gene *DapB.* The nuclei are counterstained with DAPI and the scale bar corresponds to 100 µm.

Supplementary Material 4. Supplemental Figure S4: Muscle regeneration potential declines early during the juvenile period in trout. Pictures of muscle cross-sections showing immunolabeling for myosin (blue) and tracer (red) 21 days after injury (A, B). The scale bar corresponds to 100 µm. Panels A and C, correspond to injury of 10 g trout muscle. Panels B and D, correspond to injury of 1 kg trout muscle. Panels C and D correspond to fiber size distribution in control and injured muscle for CSA up to 10,000 µm^2^. (N = 9-10, except for 10 g injured fish where only 4 fish could be analyzed). To assess the combined effects of trout weight and muscle condition (healthy vs. injured) on fiber CSA distribution, a generalized linear mixed model (GLMM) with a log-normal distribution was applied. The dependent variable was the log-transformed adjusted CSA (log(CSA + 1)). The fixed effects included muscle condition, trout weight (10 g vs. 1 kg), and their interaction (*p*<0.001 for each). A random intercept for each image was also included to account for variability between images. Model fitting and statistical significance testing were performed using the glmmTMB package (version 1.1.9) in R (version 4.3.2). Residual diagnostics were conducted to ensure model assumptions were met.

Supplementary Material 5. Supplemental Figure S5: Muscle fiber CSA, fiber density, and *pax7*^+^ cell density depend more on fish weight than on age. (A) Mean fiber CSA of trout weighing 20 g (6 and 12 months old) and 1 kg (12 months old). (B) Fiber density (number of fibers/mm^2^) in the muscle of trout weighing 20 g (aged 6 and 12 months) and 1 kg (aged 12 months). (C) Quantification of the *pax7*^*+*^ cells/mm^2^ determined by *in situ* hybridization, in muscle from trout weighing 20 g (aged 6 and 12 months) and 1 kg (aged 12 months). All statistical significances were calculated using the non-parametric Kruskal–Wallis rank test followed by the post-hoc Dunn test. Significance levels: *p<0.05, **p<0.01, ***p<0.001.

## Data Availability

The datasets used and/or analyzed during the current study are available from the corresponding author on reasonable request.

## Abbreviations

MDCs: Muscle-derived cells
MuSCs: Muscle Stem Cells
GFP: Green Fluorescent Protein
WGA: Wheat Germ Agglutinin
DAPI: 4,6-Diamidino-2- phenylindole
Pax7: Paired box 7

## References

[1] Buckingham M. Myogenic progenitor cells and skeletal myogenesis in vertebrates. Curr Opin Genet Dev. 2006;16:525–32. 10.1016/j.gde.2006.08.008. 16930987 10.1016/j.gde.2006.08.008

[2] White RB, Biérinx A-S, Gnocchi VF, Zammit PS. Dynamics of muscle fibre growth during postnatal mouse development. BMC Dev Biol. 2010;10:21. 10.1186/1471-213X-10-21. 20175910 10.1186/1471-213X-10-21 PMC2836990

[3] Wigmore PMC, Stickland NC. Muscle development in large and small pig fetuses. J Anat. 1983;137(Pt 2)(Pt 2):235–45. https://pmc.ncbi.nlm.nih.gov/articles/PMC1171817/. PMC1171817 6630038

[4] Remignon H, Gardahaut MF, Marche G, Ricard FH. Selection for rapid growth increases the number and the size of muscle fibres without changing their typing in chickens. J Muscle Res Cell Motil. 1995;16:95–102. 10.1007/BF00122527. 7622630 10.1007/BF00122527

[5] Kelly AM. Satellite cells and myofiber growth in the rat soleus and extensor digitorum longus muscles. Dev Biol. 1978;65:1–10. 10.1016/0012-1606(78)90174-4. 680348 10.1016/0012-1606(78)90174-4

[6] Johnston IA, Lee H-T, Macqueen DJ, Paranthaman K, Kawashima C, Anwar A, et al. Embryonic temperature affects muscle fibre recruitment in adult zebrafish: genome-wide changes in gene and microRNA expression associated with the transition from hyperplastic to hypertrophic growth phenotypes. J Exp Biol. 2009;212:1781–93. 10.1242/jeb.029918. 19482995 10.1242/jeb.029918

[7] Kelu JJ, Zhang J, Pipalia TG, Berthold A, Brack AS, Cheung A, et al. Muscle sparing through differential nutritional control of three muscle growth mechanisms: how zebrafish larvae deal with starvation. Dev Biol. 2026;529:254–70. 10.1016/j.ydbio.2025.10.020. 41161566 10.1016/j.ydbio.2025.10.020

[8] Kumar U, Fang C-Y, Roan H-Y, Hsu S-C, Wang C-H, Chen C-H. Whole-body replacement of larval myofibers generates permanent adult myofibers in zebrafish. EMBO J. 2024;43:3090–115. 10.1038/s44318-024-00136-y. 38839992 10.1038/s44318-024-00136-y PMC11294464

[9] Nguyen PD, Gurevich DB, Sonntag C, Hersey L, Alaei S, Nim HT, et al. Muscle stem cells undergo extensive clonal drift during tissue growth via Meox1-mediated induction of G2 cell-cycle arrest. Cell Stem Cell. 2017;21:107-119.e6. 10.1016/j.stem.2017.06.003. 28686860 10.1016/j.stem.2017.06.003

[10] Johnston IA, Strugnell G, McCracken ML, Johnstone R. Muscle growth and development in normal-sex-ratio and all-female diploid and triploid Atlantic salmon. J Exp Biol. 1999;202:1991–2016. 10393816 10.1242/jeb.202.15.1991

[11] Weatherley AH, Gill HS, Lobo AF. Recruitment and maximal diameter of axial muscle fibres in teleosts and their relationship to somatic growth and ultimate size. J Fish Biol. 1988;33:851–9. 10.1111/j.1095-8649.1988.tb05532.x.

[12] Mauro A. Satellite cell of skeletal muscle fibers. J Biophys Biochem Cytol. 1961;9:493–5. 13768451 10.1083/jcb.9.2.493 PMC2225012

[13] Bentzinger CF, Wang YX, Rudnicki MA. Building muscle: molecular regulation of myogenesis. Cold Spring Harb Perspect Biol 2012;4 10.1101/cshperspect.a008342 PMC3281568 22300977

[14] Relaix F, Montarras D, Zaffran S, Gayraud-Morel B, Rocancourt D, Tajbakhsh S, et al. Pax3 and Pax7 have distinct and overlapping functions in adult muscle progenitor cells. J Cell Biol. 2006;172:91–102. 16380438 10.1083/jcb.200508044 PMC2063537

[15] Gurevich DB, Nguyen PD, Siegel AL, Ehrlich OV, Sonntag C, Phan JMN, et al. Asymmetric division of clonal muscle stem cells coordinates muscle regeneration in vivo. Science. 2016;353:aad9969. 10.1126/science.aad9969. 27198673 10.1126/science.aad9969

[16] Berberoglu MA, Gallagher TL, Morrow ZT, Talbot JC, Hromowyk KJ, Tenente IM, et al. Satellite-like cells contribute to pax7-dependent skeletal muscle repair in adult zebrafish. Dev Biol. 2017;424:162–80. 10.1016/j.ydbio.2017.03.004. 28279710 10.1016/j.ydbio.2017.03.004 PMC5437870

[17] Seger C, Hargrave M, Wang X, Chai RJ, Elworthy S, Ingham PW. Analysis of Pax7 expressing myogenic cells in zebrafish muscle development, injury, and models of disease. Dev Dyn. 2011;240:2440–51. 10.1002/dvdy.22745. 21954137 10.1002/dvdy.22745

[18] Rallière C, Jagot S, Sabin N, Gabillard J-C. Dynamics of pax7 expression during development, muscle regeneration, and in vitro differentiation of satellite cells in the trout 2023:2023.07.19.549701. 10.1101/2023.07.19.549701. 10.1371/journal.pone.0300850 PMC11078358 38718005

[19] Relaix F, Zammit PS. Satellite cells are essential for skeletal muscle regeneration: the cell on the edge returns centre stage. Development. 2012;139:2845–56. 10.1242/dev.069088. 22833472 10.1242/dev.069088

[20] Sousa-Victor P, Gutarra S, García-Prat L, Rodriguez-Ubreva J, Ortet L, Ruiz-Bonilla V, et al. Geriatric muscle stem cells switch reversible quiescence into senescence. Nature. 2014;506:316–21. 10.1038/nature13013. 24522534 10.1038/nature13013

[21] Sousa-Victor P, Muñoz-Cánoves P. Regenerative decline of stem cells in sarcopenia. Mol Aspects Med. 2016;50:109–17. 10.1016/j.mam.2016.02.002. 26921790 10.1016/j.mam.2016.02.002

[22] Duan D, Goemans N, Takeda S, Mercuri E, Aartsma-Rus A. Duchenne muscular dystrophy. Nat Rev Dis Primers. 2021;7:1–19. 10.1038/s41572-021-00248-3. 33602943 10.1038/s41572-021-00248-3 PMC10557455

[23] Price FD, Von Maltzahn J, Bentzinger CF, Dumont NA, Yin H, Chang NC, et al. Inhibition of JAK-STAT signaling stimulates adult satellite cell function. Nat Med. 2014;20:1174–81. 10.1038/nm.3655. 25194569 10.1038/nm.3655 PMC4191983

[24] Carlson BM, Faulkner JA. Muscle transplantation between young and old rats: age of host determines recovery. Am J Physiol. 1989;256:C1262–6. 2735398 10.1152/ajpcell.1989.256.6.C1262

[25] Rescan P-Y, Rallière C, Lebret V, Fretaud M. Analysis of muscle fibre input dynamics using a myog:GFP transgenic trout model. J Exp Biol. 2015;218:1137–42. 10.1242/jeb.113704. 25657208 10.1242/jeb.113704

[26] Stickland NC. Growth and development of muscle fibres in the rainbow trout (*Salmo gairdneri*). J Anat. 1983;137(Pt 2):323–33. 6630043 PMC1171824

[27] Jagot S, Sabin N, Le Cam A, Bugeon J, Rescan P-Y, Gabillard J-C. Histological, transcriptomic and in vitro analysis reveal an intrinsic activated state of myogenic precursors in hyperplasic muscle of trout. BMC Genomics. 2018;19:865. 10.1186/s12864-018-5248-y. 30509177 10.1186/s12864-018-5248-y PMC6276237

[28] Landemaine A, Ramirez-Martinez A, Monestier O, Sabin N, Rescan P-Y, Olson EN, et al. Trout myomaker contains 14 minisatellites and two sequence extensions but retains fusogenic function. J Biol Chem. 2019;294:6364–74. 10.1074/jbc.RA118.006047. 30819805 10.1074/jbc.RA118.006047 PMC6484109

[29] Gabillard J-C, Rallière C, Sabin N, Rescan P-Y. The production of fluorescent transgenic trout to study in vitro myogenic cell differentiation. BMC Biotechnol. 2010;10:39. 10.1186/1472-6750-10-39. 20478014 10.1186/1472-6750-10-39 PMC2887378

[30] Mommsen TP. Paradigms of growth in fish. Comp Biochem Physiol B Biochem Mol Biol. 2001;129:207–19. 11399452 10.1016/s1096-4959(01)00312-8

[31] Gabillard JC, Sabin N, Paboeuf G. In vitro characterization of proliferation and differentiation of trout satellite cells. Cell Tissue Res. 2010;342:471–7. 10.1007/s00441-010-1071-8. 21086139 10.1007/s00441-010-1071-8

[32] Schindelin J, Arganda-Carreras I, Frise E, Kaynig V, Longair M, Pietzsch T, et al. Fiji: an open-source platform for biological-image analysis. Nat Methods. 2012;9:676–82. 10.1038/nmeth.2019. 22743772 10.1038/nmeth.2019 PMC3855844

[33] Pena SD, Gordon BB, Karpati G, Carpenter S. Lectin histochemistry of human skeletal muscle. J Histochem Cytochem. 1981;29:542–6. 10.1177/29.4.6166659. 6166659 10.1177/29.4.6166659

[34] Seiliez I, Froehlich JM, Marandel L, Gabillard J-C, Biga PR. Evolutionary history and epigenetic regulation of the three paralogous pax7 genes in rainbow trout. Cell Tissue Res. 2015;359:715–27. 10.1007/s00441-014-2060-0. 25487404 10.1007/s00441-014-2060-0 PMC4344431

[35] Stringer C, Wang T, Michaelos M, Pachitariu M. Cellpose: a generalist algorithm for cellular segmentation. Nat Methods. 2021;18:100–6. 10.1038/s41592-020-01018-x. 33318659 10.1038/s41592-020-01018-x

[36] Schmidt U, Weigert M, Broaddus C, Myers G. Cell Detection with Star-Convex Polygons. In: Frangi AF, Schnabel JA, Davatzikos C, Alberola-López C, Fichtinger G, editors. Med. Image Comput. Comput. Assist. Interv. – MICCAI 2018, Cham: Springer International Publishing; 2018, p. 265–73. 10.1007/978-3-030-00934-2_30.

[37] Jagot S, Babarit C, Sabin N, Rouger K, Gabillard J-C. Distinct muscle stem cell fates governing hyperplasia and hypertrophy muscle growth in fish 2026:2026.01.28.702282. 10.64898/2026.01.28.702282.

[38] Ruparelia AA, Salavaty A, Barlow CK, Lu Y, Sonntag C, Hersey L, et al. The African killifish: a short-lived vertebrate model to study the biology of sarcopenia and longevity. Aging Cell. 2023;23:e13862. 10.1111/acel.13862. 37183563 10.1111/acel.13862 PMC10776123

[39] Kimmel CB, Ballard WW, Kimmel SR, Ullmann B, Schilling TF. Stages of embryonic development of the zebrafish. Dev Dyn. 1995;203:253–310. 8589427 10.1002/aja.1002030302

[40] Brack AS, Bildsoe H, Hughes SM. Evidence that satellite cell decrement contributes to preferential decline in nuclear number from large fibres during murine age-related muscle atrophy. J Cell Sci. 2005;118:4813–21. 10.1242/jcs.02602. 16219688 10.1242/jcs.02602

[41] Chakkalakal JV, Jones KM, Basson MA, Brack AS. The aged niche disrupts muscle stem cell quiescence. Nature. 2012;490:355–60. 10.1038/nature11438. 23023126 10.1038/nature11438 PMC3605795

[42] Shefer G, Van de Mark DP, Richardson JB, Yablonka-Reuveni Z. Satellite-cell pool size does matter: Defining the myogenic potency of aging skeletal muscle. Dev Biol. 2006;294:50–66. 16554047 10.1016/j.ydbio.2006.02.022 PMC2710453

[43] Arpke RW, Shams AS, Collins BC, Larson AA, Lu N, Lowe DA, et al. Preservation of satellite cell number and regenerative potential with age reveals locomotory muscle bias. Skelet Muscle. 2021;11:22. 10.1186/s13395-021-00277-2. 34481522 10.1186/s13395-021-00277-2 PMC8418011

[44] Carlson ME, Hsu M, Conboy IM. Imbalance between pSmad3 and Notch induces CDK inhibitors in old muscle stem cells. Nature. 2008;454:528–32. 10.1038/nature07034. 18552838 10.1038/nature07034 PMC7761661

[45] Conboy IM, Conboy MJ, Wagers AJ, Girma ER, Weissman IL, Rando TA. Rejuvenation of aged progenitor cells by exposure to a young systemic environment. Nature. 2005;433:760–4. 10.1038/nature03260. 15716955 10.1038/nature03260

[46] Lukjanenko L, Jung MJ, Hegde N, Perruisseau-Carrier C, Migliavacca E, Rozo M, et al. Loss of fibronectin from the aged stem cell niche affects the regenerative capacity of skeletal muscle in mice. Nat Med. 2016;22:897–905. 10.1038/nm.4126. 27376579 10.1038/nm.4126 PMC5467443

[47] Brack AS, Rando TA. Intrinsic changes and extrinsic influences of myogenic stem cell function during aging. Stem Cell Rev. 2007;3:226–37. 10.1007/s12015-007-9000-2. 17917136 10.1007/s12015-007-9000-2

[48] Tavakoli S, Garcia V, Gähwiler E, Adatto I, Rangan A, Messemer KA, et al. Transplantation-based screen identifies inducers of muscle progenitor cell engraftment across vertebrate species. Cell Rep. 2023;42:112365. 10.1016/j.celrep.2023.112365. 37018075 10.1016/j.celrep.2023.112365 PMC10548355

[49] Rowlerson A, Radaelli G, Mascarello F, Veggetti A. Regeneration of skeletal muscle in two teleost fish: *Sparus aurata* and *Brachydanio rerio*. Cell Tissue Res. 1997;289:311–22. 9211834 10.1007/s004410050878

[50] Perelló-Amorós M, Otero-Tarrazón A, Jorge-Pedraza V, García-Pérez I, Sánchez-Moya A, Gabillard J-C, et al. Myomaker and Myomixer characterization in Gilthead Sea Bream under different myogenesis conditions. Int J Mol Sci. 2022;23:14639. 10.3390/ijms232314639. 36498967 10.3390/ijms232314639 PMC9737248

[51] Otero-Tarrazón A, Perelló-Amorós M, Jorge-Pedraza V, Moshayedi F, Sánchez-Moya A, García-Pérez I, et al. Muscle regeneration in gilthead sea bream: Implications of endocrine and local regulatory factors and the crosstalk with bone. Front Endocrinol. 2023;14:1101356. 10.3389/fendo.2023.1101356. 10.3389/fendo.2023.1101356 PMC9899866 36755925

[52] Froehlich JM, Fowler ZG, Galt NJ, Smith DL Jr, Biga PR. Sarcopenia and piscines: the case for indeterminate-growing fish as unique genetic model organisms in aging and longevity research. Front Genet. 2013;4:159. 10.3389/fgene.2013.00159. 23967015 10.3389/fgene.2013.00159 PMC3743216

