# Supplementary material for: Declining muscle hyperplasia in juvenile trout is associated with a significant impairment of the supportive function of the myogenic progenitor niche
Source: Skelet Muscle. 2026 Jun 8;16:30. doi: 10.1186/s13395-026-00431-8 (PMC13474579; doi:10.1186/s13395-026-00431-8)
Supplement: Supplementary file 1 [file 13395_2026_431_MOESM1_ESM.pdf]

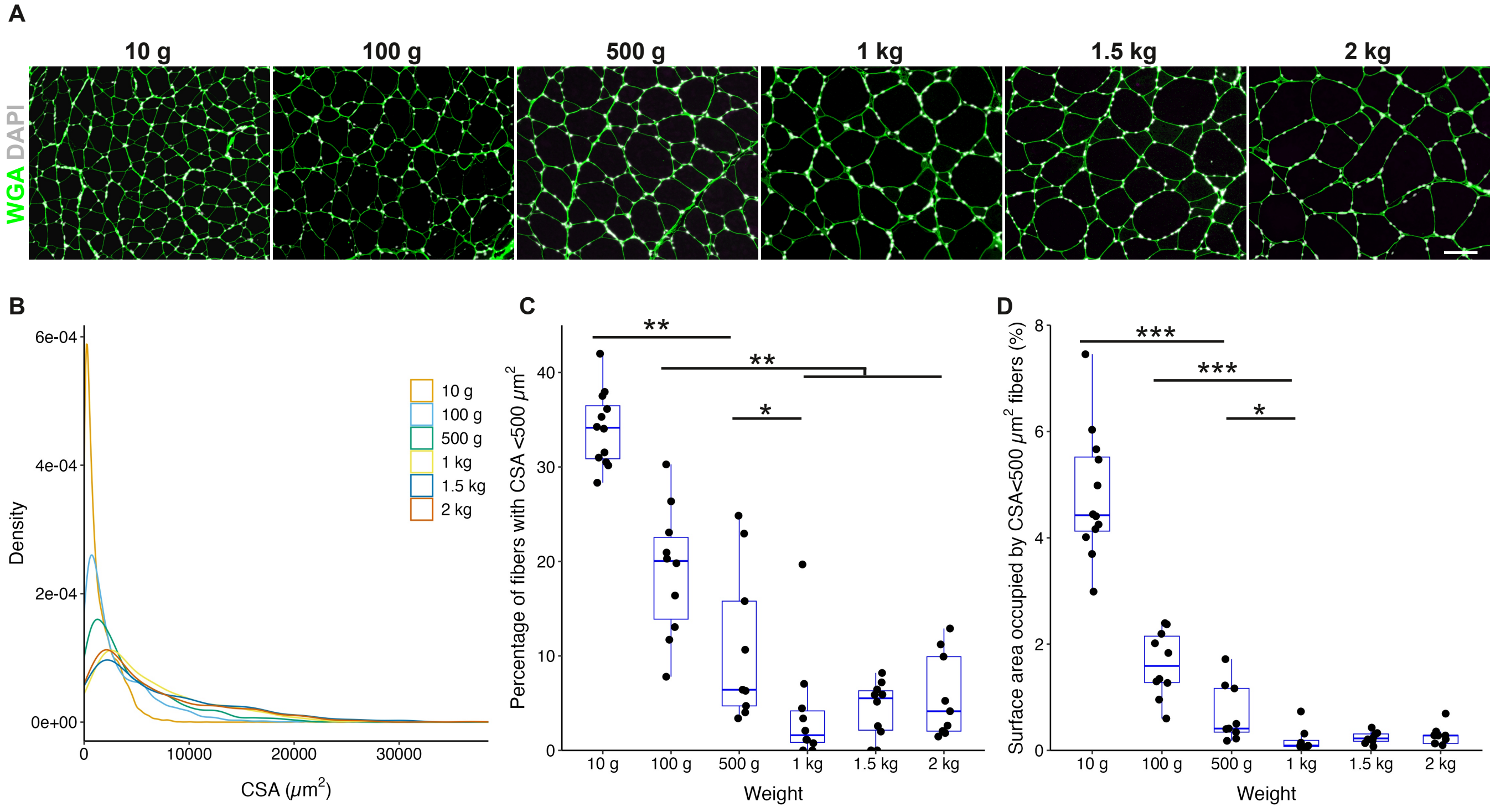

A
10 g
100 g
500 g
1 kg
1.5 kg
2 kg
WGA DAPI
B
Density
6e-04
4e-04
2e-04
0e+00
0
10000
20000
30000
CSA (μm²)
10 g
100 g
500 g
1 kg
1.5 kg
2 kg
C
Percentage of fibers with CSA <500 μm²
**
**
*
40
30
20
10
0
10 g
100 g
500 g
1 kg
1.5 kg
2 kg
Weight
D
Surface area occupied by CSA<500 μm² fibers (%)
***
***
*
8
6
4
2
0
10 g
100 g
500 g
1 kg
1.5 kg
2 kg
Weight
