# Supplementary material for: Declining muscle hyperplasia in juvenile trout is associated with a significant impairment of the supportive function of the myogenic progenitor niche
Source: Skelet Muscle. 2026 Jun 8;16:30. doi: 10.1186/s13395-026-00431-8 (PMC13474579; doi:10.1186/s13395-026-00431-8)
Supplement: Supplementary file 2 [file 13395_2026_431_MOESM2_ESM.pdf]

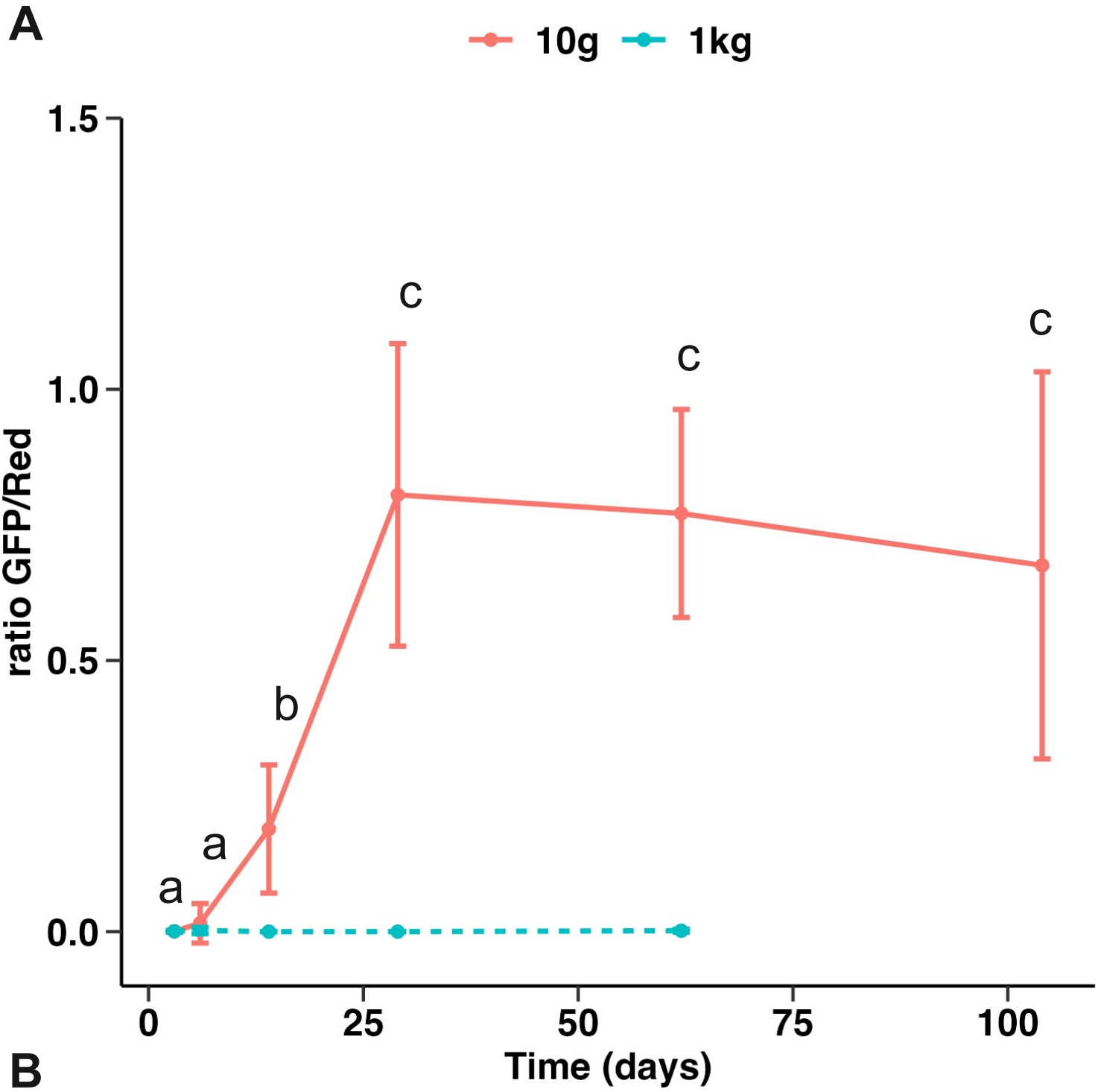
A
10g
1kg
1.5
1.0
0.5
0.0
ratio GFP/Red
c
c
c
b
a
a
0
25
50
75
100
Time (days)

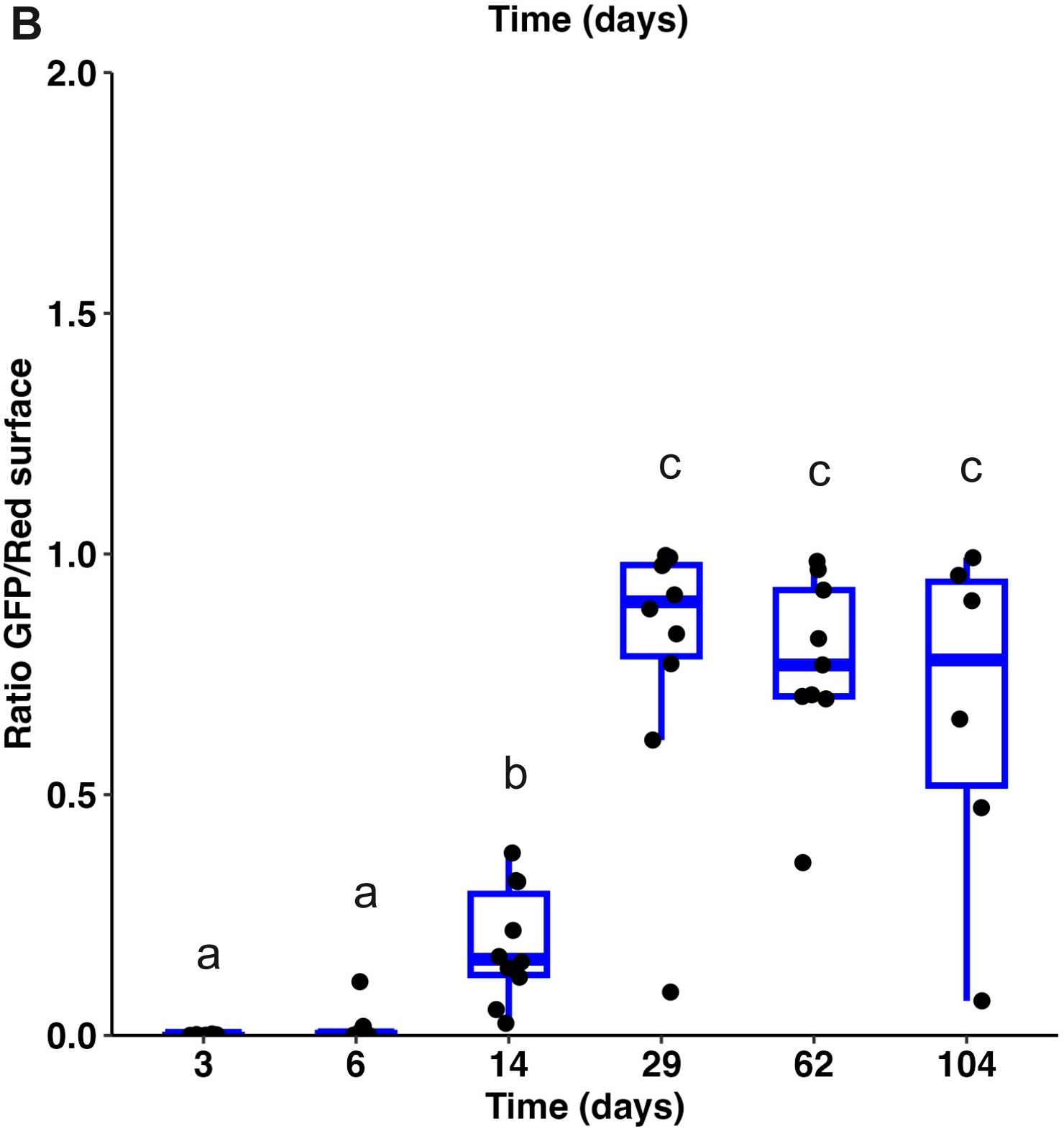
B
2.0
1.5
1.0
0.5
0.0
Ratio GFP/Red surface
c
c
c
b
a
a
3
6
14
29
62
104
Time (days)
