# Supplementary material for: Declining muscle hyperplasia in juvenile trout is associated with a significant impairment of the supportive function of the myogenic progenitor niche
Source: Skelet Muscle. 2026 Jun 8;16:30. doi: 10.1186/s13395-026-00431-8 (PMC13474579; doi:10.1186/s13395-026-00431-8)
Supplement: Supplementary file 3 [file 13395_2026_431_MOESM3_ESM.pdf]

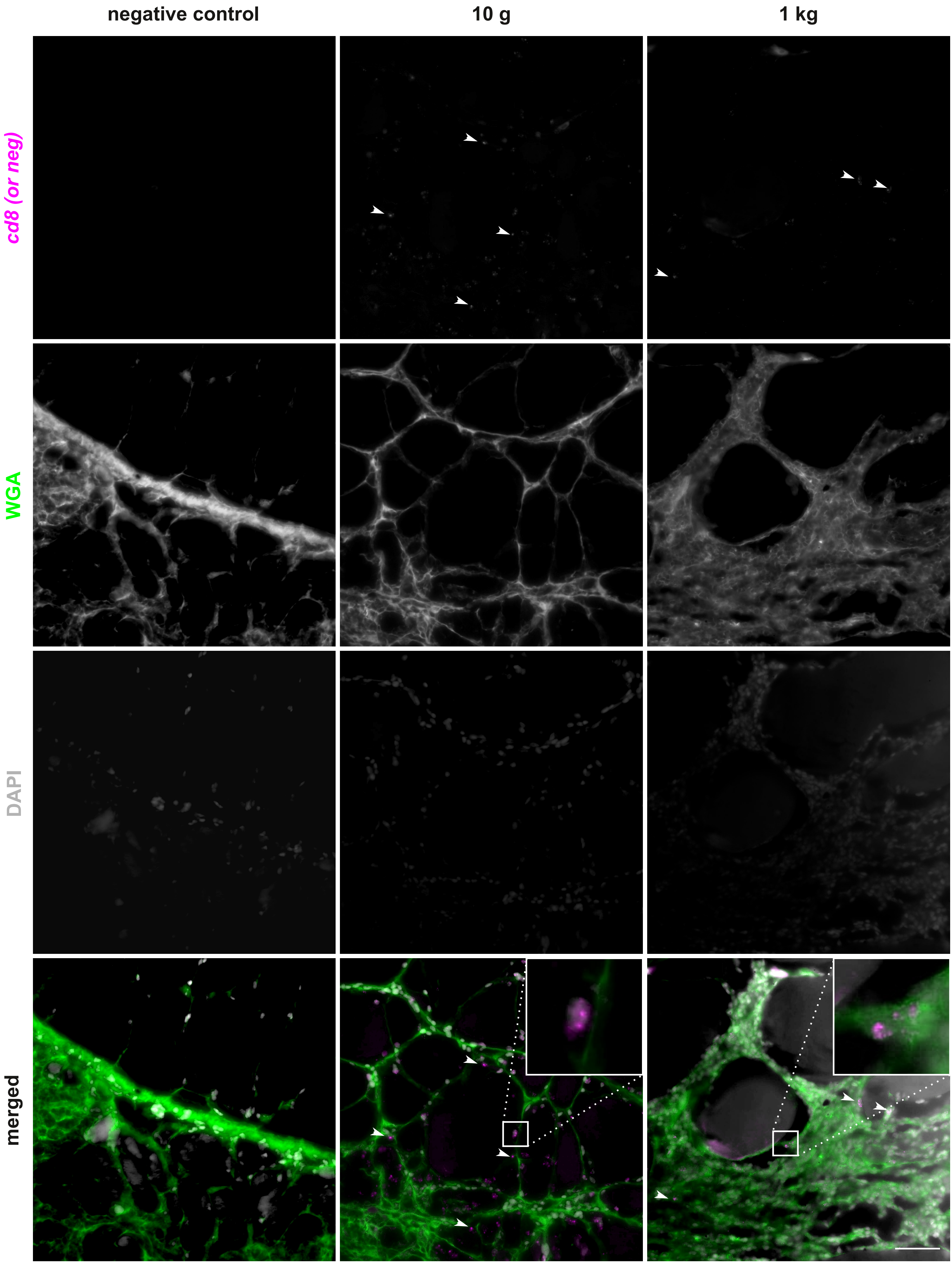

negative control
10 g
1 kg
cd8 (or neg)
WGA
DAPI
merged
