# Supplementary material for: Declining muscle hyperplasia in juvenile trout is associated with a significant impairment of the supportive function of the myogenic progenitor niche
Source: Skelet Muscle. 2026 Jun 8;16:30. doi: 10.1186/s13395-026-00431-8 (PMC13474579; doi:10.1186/s13395-026-00431-8)
Supplement: Supplementary file 4 [file 13395_2026_431_MOESM4_ESM.pdf]

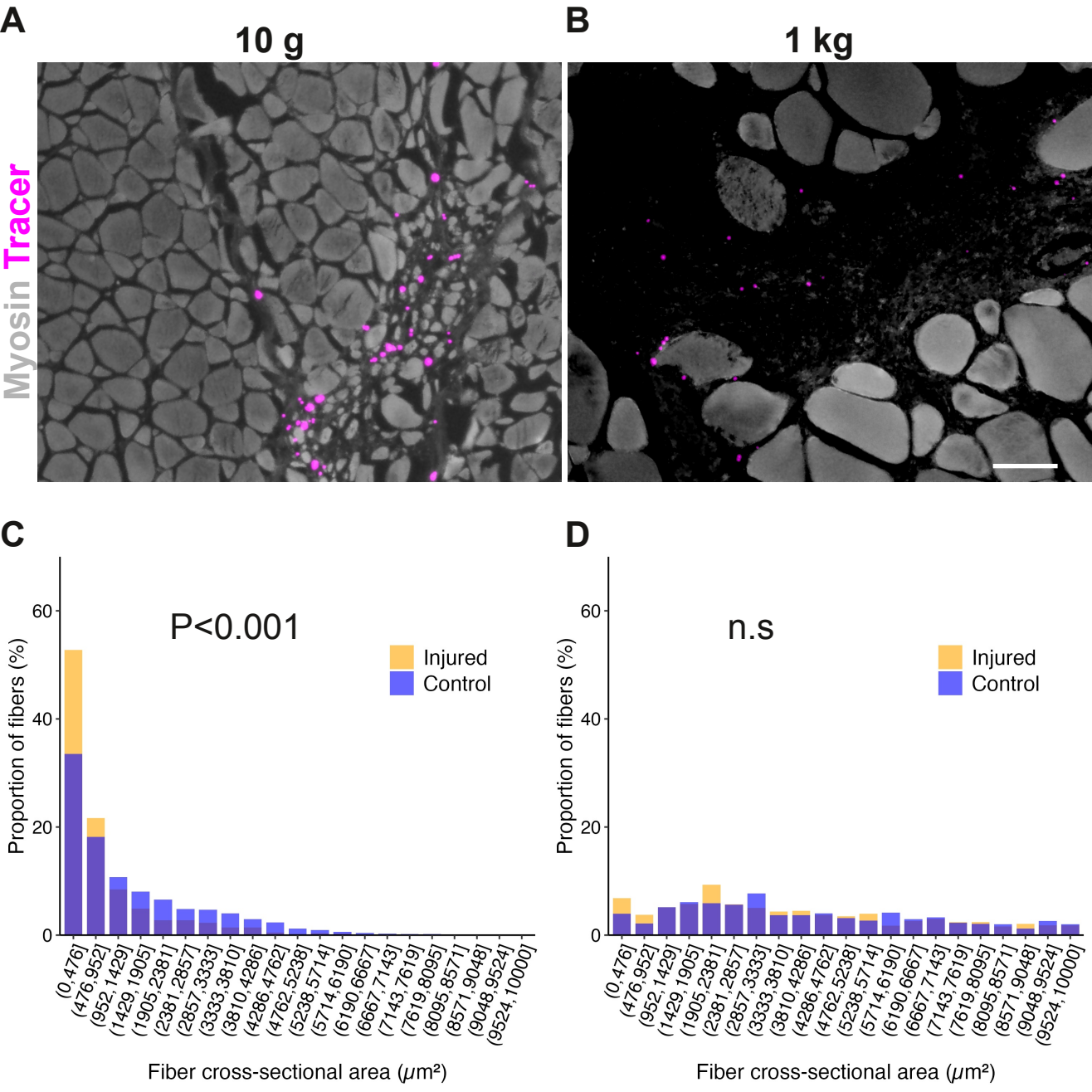

A
10 g
B
1 kg
Myosin Tracer
C
P<0.001
Injured
Control
Proportion of fibers (%)
Fiber cross-sectional area (μm²)
D
n.s
Injured
Control
Proportion of fibers (%)
Fiber cross-sectional area (μm²)
0
20
40
60
(0,476]
(476,952]
(952,1429]
(1429,1905]
(1905,2381]
(2381,2857]
(2857,3333]
(3333,3810]
(3810,4286]
(4286,4762]
(4762,5238]
(5238,5714]
(5714,6190]
(6190,6667]
(6667,7143]
(7143,7619]
(7619,8095]
(8095,8571]
(8571,9048]
(9048,9524]
(9524,10000]
