# Supplementary material for: Declining muscle hyperplasia in juvenile trout is associated with a significant impairment of the supportive function of the myogenic progenitor niche
Source: Skelet Muscle. 2026 Jun 8;16:30. doi: 10.1186/s13395-026-00431-8 (PMC13474579; doi:10.1186/s13395-026-00431-8)
Supplement: Supplementary file 5 [file 13395_2026_431_MOESM5_ESM.pdf]

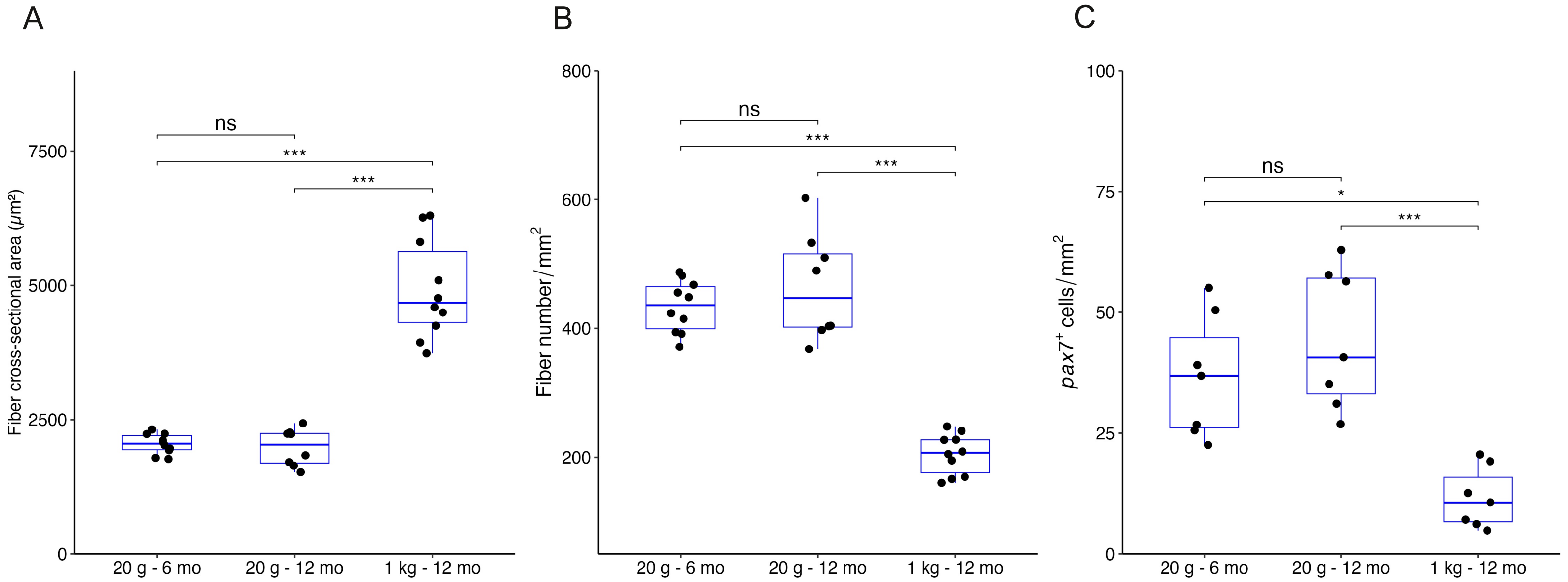

A
B
C
ns
***
***
7500
5000
2500
0
Fiber cross-sectional area (μm²)
20 g - 6 mo
20 g - 12 mo
1 kg - 12 mo
800
600
400
200
Fiber number/mm2
100
75
50
25
*
pax7+ cells/mm2
